# Proton Radiation Therapy for Nasopharyngeal Cancer Patients: Dosimetric and NTCP Evaluation Supporting Clinical Decision

**DOI:** 10.3390/cancers14051109

**Published:** 2022-02-22

**Authors:** Alessandro Vai, Silvia Molinelli, Eleonora Rossi, Nicola Alessandro Iacovelli, Giuseppe Magro, Anna Cavallo, Emanuele Pignoli, Tiziana Rancati, Alfredo Mirandola, Stefania Russo, Rossana Ingargiola, Barbara Vischioni, Maria Bonora, Sara Ronchi, Mario Ciocca, Ester Orlandi

**Affiliations:** 1 Radiotherapy Department, Center for National Oncological Hadrontherapy (CNAO), 27100 Pavia, Italy; silvia.molinelli@cnao.it (S.M.); eleonora.rossi@cnao.it (E.R.); giuseppe.magro@cnao.it (G.M.); stefania.russo@cnao.it (S.R.); rossana.ingargiola@cnao.it (R.I.); barbara.vischioni@cnao.it (B.V.); maria.bonora@cnao.it (M.B.); sara.ronchi@cnao.it (S.R.); mario.ciocca@cnao.it (M.C.); ester.orlandi@cnao.it (E.O.); 2 Radiotherapy Department, Fondazione IRCCS Istituto Nazionale dei Tumori di Milano (INT), 20133 Milan, Italy; anna.cavallo@istitutotumori.mi.it (A.C.); emanuele.pignoli@istitutotumori.mi.it (E.P.); tiziana.rancati@istitutotumori.mi.it (T.R.); alfredo.mirandola@cnao.it (A.M.)

**Keywords:** proton therapy, nasopharyngeal cancer, NTCP, model-based selection

## Abstract

**Simple Summary:**

Radiotherapy is the cornerstone of treatment of nasopharyngeal cancer, in different settings with or without chemotherapy. This role has been recently strengthened by the introduction of proton therapy, as a radiation treatment option for head and neck cancer, obtaining improved plans with a reduced dose to organs-at-risk. Definition of strategies to identify patients who would benefit the most from proton therapy in terms of reduced toxicity is highly desirable, due to limited availability and higher costs of this treatment option. Two parallel working pipelines were depicted in this study for nasopharyngeal cancer patients. The introduction of a synthetic index describing the overall expected reduction in toxicities in the head and neck region with proton therapy was supported by the application of the well-established model-based selection methodology, relative to the same patient cohort. Based on this analysis, the fraction of nasopharyngeal cancer patients expected to receive a benefit with proton therapy was in line with the Dutch experience for the head and neck cancer population.

**Abstract:**

(1) Background: we proposed an integrated strategy to support clinical allocation of nasopharyngeal patients between proton and photon radiotherapy. (2) Methods: intensity-modulated proton therapy (IMPT) plans were optimized for 50 consecutive nasopharyngeal carcinoma (NPC) patients treated with volumetric modulated arc therapy (VMAT), and differences in dose and normal tissue complication probability (ΔNTCPx-p) for 16 models were calculated. Patient eligibility for IMPT was assessed using a model-based selection (MBS) strategy following the results for 7/16 models describing the most clinically relevant endpoints, applying a model-specific ΔNTCPx-p threshold (15% to 5% depending on the severity of the complication) and a composite threshold (35%). In addition, a comprehensive toxicity score (CTS) was defined as the weighted sum of all 16 ΔNTCPx-p, where weights follow a clinical rationale. (3) Results: Dose deviations were in favor of IMPT (ΔD_mean_ ≥ 14% for cord, esophagus, brainstem, and glottic larynx). The risk of toxicity significantly decreased for xerostomia (−12.5%), brain necrosis (−2.3%), mucositis (−3.2%), tinnitus (−8.6%), hypothyroidism (−9.3%), and trismus (−5.4%). There were 40% of the patients that resulted as eligible for IMPT, with a greater advantage for T3–T4 staging. Significantly different CTS were observed in patients qualifying for IMPT. (4) Conclusions: The MBS strategy successfully drives the clinical identification of NPC patients, who are most likely to benefit from IMPT. CTS summarizes well the expected global gain.

## 1. Introduction

Nasopharyngeal carcinoma (NPC) is a rare tumor with unique epidemiological and histological features, commonly affecting Asian countries and rarely European ones [1,2]. Radiotherapy (RT) is the cornerstone of the treatment, with or without chemotherapy (CHT) in different settings (concurrent and or adjuvant and or neoadjuvant) according to disease stage and EBV-plasma load [3]. So far, IMRT represents the current standard RT technique, having allowed to significantly reduce late toxicities such as xerostomia, trismus, temporal lobe necrosis, neurological injury, and hearing impairment, compared with older RT techniques, without jeopardizing clinical outcome [3]. However, toxicity rates are still relevant, especially for advanced clinical stages, with substantial effects on quality of life (QOL) [4,5,6,7]. The key role played by RT has been recently strengthened by the introduction of proton therapy (PT) as a RT treatment option for head and neck cancer (HNC), including NPC [8]. In particular, intensity-modulated proton therapy (IMPT) has the possibility to reduce radiation-induced side-effects to organs-at-risk (OARs) while guaranteeing highly conformal coverage of the target compared to advanced photon-based RT techniques, such as volumetric modulated arc therapy (VMAT) [9]. Planned comparison studies between IMPT and IMRT techniques have suggested that lower (mean) doses can be delivered to several organs at risk (OARs) in patients with NPC [10,11,12,13], without significant difference in target coverage, conformity, or homogeneity indexes [10,14]. A few small studies have shown a benefit in the clinical outcome when IMPT and IMRT were compared [11,15]. Nevertheless, due to the higher costs and relatively limited availability of IMPT with respect to conventional photon-based RT, PT should be reserved for patients that are likely to benefit the most in terms of toxicity risk reduction. Recently, model-based clinical evaluations have been proposed as valid evidence-based methods alternative to randomized controlled trials [16]. Model-based selection methodology is an approach developed by Langendijk et al. [17] primarily regarding HNC radiotherapy RT. The dose reduction to relevant OARs, resulting from a plan comparison between protons and photons, is translated into a clinically relevant benefit, estimated in terms of reduced risk of side effects as assessed by normal tissue complication probability (NTCP) models. The individual patient is eligible for proton therapy if the difference in the predicted risks (protons vs. photons ΔNTCP) is larger than a defined clinically relevant threshold, e.g., 10% for a grade 2 toxicity, which represents the minimal potential benefit to qualify the patient for PT [18]. The burden of late toxicity for NPC patients primarily consists in xerostomia, dysphagia, trismus, temporal lobe necrosis, neurological injury, and hearing impairment, being quite different from the general HNC population [4,5,6,7,19]. In addition, acute grade ≥ 3 oropharyngeal mucositis affects up to 30% of NPC patients during IMRT plus chemotherapy [20].

The aim of this work was to investigate a combined method to estimate the proportion of NPC patients that may benefit from PT. We performed an in silico planning comparison on a cohort of NPC patients already treated with VMAT and chemotherapy. We applied the model-based selection (MBS) methodology referring to a limited set of toxicity endpoints of major clinical relevance for NPC population. A parallel selection workflow was proposed, in which the ΔNTCP results from our extensive analysis were combined into a concise index, i.e., comprehensive toxicity score (CTS). As a final step, the two approaches were related by identifying a CTS value for PT eligibility for the considered cohort based on the results of the MBS analysis.

## 2. Materials and Methods

We performed a retrospective comparative study on a cohort of 50 consecutive non metastatic NPC patients (age at the time of treatment [median, range]: [51, 24–72]) treated between 2016 and 2019 with curative VMAT with or without chemotherapy at Fondazione IRCCS Istituto Nazionale dei Tumori, Milan, Italy (median follow-up, range: [22, 5–39] months). These patients were included in the MICROLEARNER study, which aimed to investigate the role of salivary microbiome in determining toxicity in the HNC population (ClinicalTrials.gov Identifier: NCT03294122). An inter-institutional agreement to share and transfer data was signed. Ethical approval by the institutional review board was obtained for this study (INT 72/21).

Clinical details of target volume delineation and VMAT planning and delivery procedures have been previously reported [21]. Briefly, VMAT plans consisted of 6 MV two-four coplanar arcs and dose was computed using an Anisotropic Analytical Algorithm with a dose grid resolution of 2.5 mm^3^. A prescription dose of 69.96 Gy (RBE) was administered in 33 fractions to the high-dose planning target volume (PTVHD) with a simultaneous integrated boost regimen. Two different low dose (LD) levels to be delivered to the PTVLD were defined: 56.1 and 59.4 Gy(RBE) for, respectively, 12 (24%) and 10 (20%) patients; while 28 (56%) patients received a three dose levels prescription: 69.96, 59.4, 56.1 Gy(RBE) to PTVHD, intermediate dose (PTVID), and PTVLD, respectively [22]. PTVHD dose coverage was optimized to obtain at least 95% of prescription dose to 100% of the volume (V100%), except in case of proximity to critical neurological structures. In those cases, priority to the sparing of the critical OARs was given, accepting a 95% PTV coverage [23].

OARs dose constraints followed an internal protocol adhering to QUANTEC indications [23,24] (Table S3 in Supplementary Materials).

A pencil beam scanning (PBS) IMPT plan was optimized for each patient with the Monte Carlo (MC) engine of the RayStation treatment planning system (TPS) v.8B (Raysearch laboratories AB, Stockholm, Sweden) with four coplanar beams: two anterior oblique beams with gantry angles of 45° and 315° and two contralateral beams with gantry angles of 90° and 270° for treating the nasopharynx and bilateral neck. The proton fixed horizontal beamline available at our facility was used for plan optimization, simulating a gantry geometry. All plans were optimized with a lateral scan step of 3 mm and an energy step corresponding to 2 mm in depth. RBE was set to a constant value of 1.1 for RBE-weighted dose calculation, following the current recommendations for clinical practice [25]. The caudal part of the target was covered by the anterior oblique beams only, in order to avoid the shoulders and prevent range variations due to patient setup uncertainties. The limited computational power hindered the use of our standard recipe for robust optimization of HNC patients with isotropic 2-mm set-up and 3% range uncertainty, based on our daily image guidance protocol [26]. For this in-silico study, IMPT plans were optimized on the PTV following the same goals of the VMAT plans.

The following quantities were used for target coverage evaluation: doses to 99%, 95%, 50%, and 1% of the volume (D99%, D95%, D50%, and D1%), the conformity index CI (defined as the ratio of the volume of PTV receiving a dose equal or higher than 95% of the prescription dose (𝑉95%) and the PTV volume) and the homogeneity index HI (defined as HI = (D2%−D98%)/D50%)). PTVHD and subtracted target volumes PTVID-HD, PTVLD-ID were considered for the analysis. The average difference between IMPT and VMAT plans, over the patient cohort, of all dose quantifiers was then calculated. For OARs, the following dose evaluator were used: D99%, D50%, D2%, D1%, and average dose (D_avg_). We also calculated the integral dose [Gy(RBE) × cm^3^], i.e., the mean dose to the patient’s (CT-scanned) body volume times the body volume, assuming no radiation exposure of the non-scanned body.

After an extensive review of the literature, sixteen NTCP models were used in this study (Table 1). Selection was based on: (1) a focus on NPC-specific toxicities based on clinical experience; (2) only studies developing, validating, or applying NTCP models, in which parameters for NTCP evaluation were made available by the authors. These NTCP-models resulted equally distributed between levels 3 (*n* = 8) and 4 (*n* = 8), according to the classification for level of evidence proposed by Stieb et al. [27] with reference to the TRIPOD statement [28] and NVRO (Nederlandse Vereniging voor Radioterherapie en Oncologie) [29]. CTS values were calculated based on this extensive models selection, summarizing the overall expected benefit in terms of reduced toxicities with IMPT for NPC patients. Among this group, seven NTCP models were identified as of major clinical relevance to be used in the MBS methodology. These models satisfied at least one of the following requirements: (a) predict grade ≥ II or higher late toxicity; (b) reflect a clinically crucial benefit in terms of toxicity reduction and quality of life improvement for NPC patients. In particular, the model predicting brain necrosis ≥ grade II was proton-derived [30], while models for grade II–IV dysphagia and moderate to severe xerostomia are the ones agreed on in the Netherlands for selecting patient for proton therapy [31]. NTCP models for radiation-induced ocular toxicity, grade > IV visual acuity loss [32] and trismus assessed as jaw-opening < 35 mm [33] were photon-derived.

At the population level, the average differences of NTCP values between VMAT and IMPT (ΔNTCPx-p) for each endpoint were calculated and tested with a two-sided Wilcoxon signed-rank test for paired samples. We set the level of statistical significance at 0.05. For all patients, the CTS was estimated as the weighted sum (see Equation (1)) of the differences of NTCP values between VMAT and IMPT for each of the sixteen selected models, with weights $w_{i}$, defining a scale of clinical relevance, as reported in Table 1.

(1) $$\overset{16}{\underset{i=1}{\sum }}w_{i}\;\left(NTCP_{X}-NTCP_{p}\right)$$

Equation (1): Comprehensive toxicity score (CTS) definition.

For each endpoint, specific thresholds were set to quantify the required minimal gain for patient indication to PT (Table 1). Patients qualified for PT if ΔNTCPx-p for a single endpoint was larger than the corresponding threshold or if the summed expected risk reduction (Σ ΔNTCPx-p) was ≥35%. These values refer to the National Indication Protocol for Particle Therapy (NIPP) [43], which suggests 10% and 5% thresholds, respectively, for grade ≥ II and grade ≥ III side effects. For xerostomia and mucositis we increased the threshold until 15% to account for the relatively lower detrimental impact on the QOL for xerostomia, and for the higher potential for recovery following mucositis. The composite threshold of 35% accounts for the number of models considered, assuming an ideal concomitant 5% variation for each of the 7 models. Patients were dichotomized into two groups: one including patients that met the eligibility criteria for PT and the other with the remaining patients according to MBS methodology. Then, differences in average CTS values between the two groups was assessed by two-sided Wilcoxon signed-rank test with a significance level of 0.05. Eligibility to PT and CTS values were investigated for each patient subgroup obtained after cohort stratification, respectively by primary tumor staging (T1–T2 and T3–T4) and nodal involvement (N0, N1, and N2–N3) (Table 2). In addition, we carried out an in-silico evaluation of the influence of patient’s age on proton eligibility, by defining weaker requirements for younger patients (e.g., <40 years old) in consideration of the longer life expectancy [44] (see Table 1). Assuming that patient’s age did not affect plan optimization strategy and criteria, the two sets of threshold values (standard vs. younger) were both applied to the full patient cohort. For each scenario, differences in average CTS values between eligible and not eligible patients were tested for significance (*p* < 0.05).

The NTCP analysis was carried out with a simple in-house software: a Python script for extraction and calculation of plan parameters coupled with a Matlab (The MathWorks, Inc., Natick, MA, USA) tool for equivalent uniform dose (EUD) and NTCP computation.

## 3. Results

Clinical characteristics for the investigated cohort are shown in Table 2. For all plans, PTVHD D99% median values agreed within 1σ ((67.5 ± 0.2) Gy(RBE) and (68.0 ± 0.6) Gy(RBE) for IMPT and VMAT, respectively). D1% showed no significant difference ((70.7 ± 2.9) Gy(RBE) versus (71.3 ± 3.2) Gy(RBE) for IMPT and VMAT, respectively). There was no significant difference in HI values for PTVHD, PTVID-HD, and PTVLD-ID. The CI was lower for IMPT plans (−10.9%). In the same way, IMPT allowed a reduction of 45% in the integral dose delivered to the patient. Complete results were reported in Tables S1 and S2 in the Supplementary Materials. The dosimetric comparison showed that PT improved.

**Figure 1:**
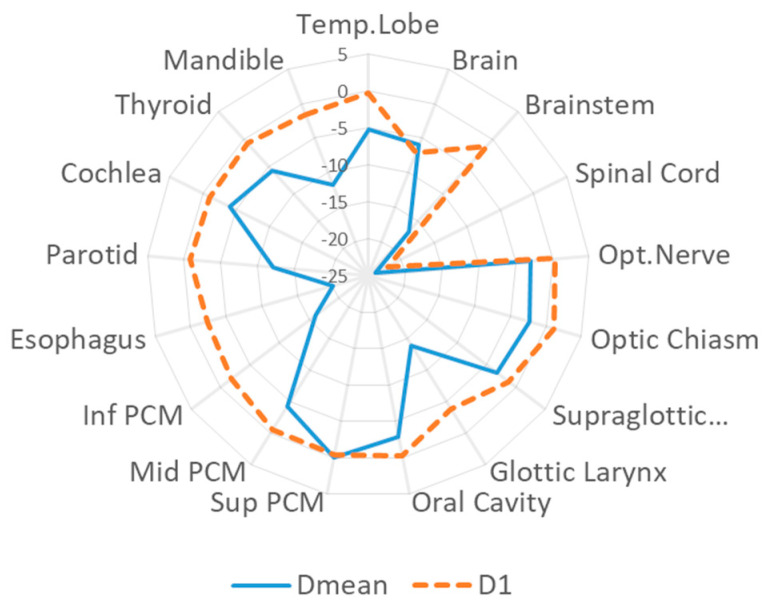
ΔDMean, ΔD1 [Gy(RBE)] between IMPT and VMAT plans for the analyzed OARs.

**Figure 2:**
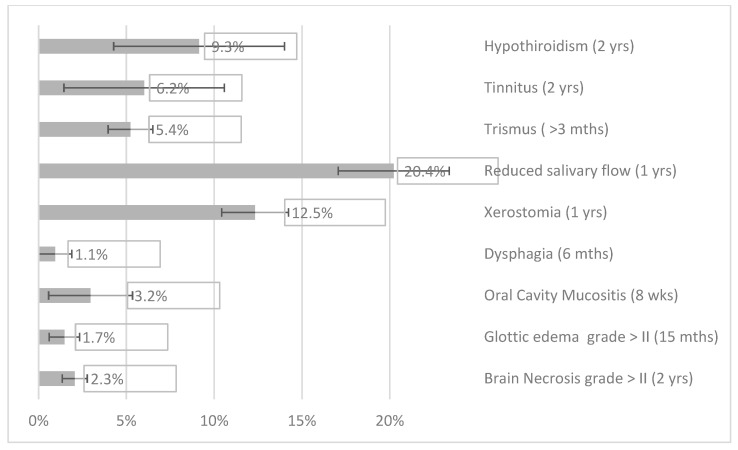
ΔNTCPx-p for the considered models. Missing models in the graph showed ΔNTCPx-p comparable to zero and were not plotted.

OARs sparing in the low-to-middle dose region for OARs close to the target (e.g., parotid glands, mandible, brainstem) while D1% values decreased for OARs located few centimeters far from the PTV, e.g., spinal cord and brain. Inferior and mid-pharyngeal constrictor muscles (PCMs) could be spared; while superior PCMs were often included in the target (see Figure 1). A comprehensive analysis of the selected dose-volume evaluation criteria was reported in Table S3 in Supplementary Materials. Figure 2 reports NTCP differences between VMAT and IMPT for the selected endpoints and models. Variations were statistically significant (*p* < 0.05) for most of the models, except for the temporal lobe infarction, radiation-induced ocular toxicity, visual loss, swallowing, and aspiration.

According to the application of the MBS strategy to our cohort, 20 patients (40%) would benefit from proton therapy (see Table 3), with, respectively, 18 (36%) and 10 (20%) patients meeting the single or the composite threshold criteria. For five patients the criteria on a single ΔNTCPx-p was fulfilled, while eight patients satisfied both one single and the composite criteria. Passing rates for each endpoint are shown in Table 3 as well. Proportion of patients eligible for PT varied noticeably using the threshold values selected for younger patients (84%).

For patients eligible for proton therapy an average CTS of 6.3 ± 1.4 was calculated, while a value of 3.5 ± 1.4 resulted for the non-eligible cohort. The difference between the two groups was statistically significant (*p* < 0.01). CTS values relative to the subgroups obtained after tumor staging stratification was reported in Table S4 of the Supplementary Materials. Significant differences between eligible and non-eligible cohort was verified (*p* < 0.05) except for the N0 subgroup (*p* = 0.09).

## 4. Discussion

Clinical decisions need to be supported by evidence-based studies, when long-term treatment outcomes are not available. In particular, in the analysis presented, we investigated a clinical strategy to guide NPC patient’s indication to PT. Our decision-making model was primarily focused on the expected reduction in grade ≥ II toxicities, which were considered clinically relevant to the patient’s benefit (xerostomia, dysphagia, brain necrosis, trismus, and optical pathways damage) and not treatable with replacement therapy (e.g., hypothyroidism and mucositis). Then, we weighted the severity of side effects according to patient’s age in consideration of the fact that younger patients generally tolerate therapy better, but they may experience a more prolonged impact on their QOL from toxicity compared to an older population.

Recently, Tambas et al. [43] reported their experience with the model-based approach for selecting patients with HNC for PT. Patients qualified for PT had more locally advanced diseases, usually pharyngeal tumors. With this approach, 35% of patients with HNC were referred to PT based on a ΔNTCP value higher than the selected threshold (ΔNTCP ≥ 10–5% for grade II–III side effects, respectively), mainly for dysphagia and xerostomia. However, in that study, a few patients had NPC. For our cohort, 20 (40%) out of 50 patients, came out to be eligible for PT, in line with the Dutch experience on the HNC population [43]. In our group, patient eligibility was determined based on a reduced risk of xerostomia (12 patients, 24%), brain necrosis (4 patients, 8%), trismus and mucositis (3 patients, 6%), and dysphagia (1 patient, 2%). PCMs, in particular superior ones, were always totally included in the CTVs and then PTVs in most cases, accordingly target volumes definition for this HNC subsite. In addition, late swallowing impairment in NPC patients is also related to cranial nerves injury (i.e., hypoglossal nerve [45]), this strongly limiting the potential gain in dysphagia related toxicities and underlines the need to build and validate appropriate models. The relative importance of brain-necrosis reflects the characteristics of the dose-response model, which was specifically designed for extracranial tumors placing a higher weight to partially irradiated volumes [30]. In other words, the used model estimates for the first time the probability of radiation necrosis based on the volumetric dose distribution in patients who underwent proton therapy. There was no gain in the risk of visual acuity loss and induced ocular toxicity, because the maximum dose constraint was below the tolerance dose for the selected endpoints and organs overlap with the PTV rarely occurs for NPC tumors. Patients with locally advanced cancers (T3–T4), where the target was often in proximity or overlapping with OARs (parotid glands, oral cavity, PCMs, and cochlea) will benefit more from proton therapy as also reported by Tambas et al. [43] (Figure S1 in the Supplementary Materials).

The NTCP MBS approach for patient selection has been successfully employed in clinical settings [18]. In this in-silico evaluation, we tested for consistency in the decision-making process a comprehensive scoring method, which accounts for a larger scenario of toxicity indicators. This came on the heels of the comprehensive toxicity risk profiling proposed by Van den Bosch et al. [46]. The CTS is a synthetic index, easy to estimate, promptly adaptable to upcoming NTCP models. It could not be used per se initially and will be subjected to further clinical investigations. To validate eligibility to PT in terms of CTS, the calculated values were therefore related to the results of the application of the MBS methodology. CTS might be refined to represent a patient-specific synthetic index of the expected global gain in terms of reduced toxicity on a larger population-based study.

The risk that the sum of several small NTCP differences might result in a substantial gain cannot be ruled out, but weights’ definition, i.e., the sum of the weights relative to the models with weight < 0.07 (11 out of 16) is ¼ of the total, reduced significantly this possibility. Our paper showed the highest benefit for protons in terms of NTCPs was for patients staged T3–T4, due to the higher dose levels to the nearby OARs such as brain, optical pathways, cochlea, and superior PCM, thus at higher risk of developing toxicities. The importance of the staging disease emerged in the paper by Tambas et al. [43]: among 227 patients with different HNC subsites, the selection rate was higher for patients with advanced disease, other than pharyngeal tumors, and/or baseline complaints. The model-based selection is time-consuming and requires considerable resources. In this scenario, clinical considerations could help to easily qualify patients eligible for protons.

One of the limitations of this study is the use of a PTV-based approach for IMPT plans optimization. Robust optimization of NPC PT plans is highly demanding in terms of computational power for several factors, e.g., large target volume, high number of involved OARs, multi-beam geometry, large number of robustness scenarios to be calculated. Stuschke et al. [47] showed that the expected reduction in NTCP values for OAR for HNC patients could potentially be greater using robust optimization than a PTV-based approach, due to reduced dose in the regions of the previous overlap between PTV and OAR. Nevertheless, a detriment in near-to-maximum OAR doses could derive from a less conformal target coverage (i.e., when robust optimization objectives on CTV are introduced) [48]. Unfavorable dosimetric deviations, with respect to our results, are therefore expected to concentrate in the near-to-maximum OAR doses, thus translating into minor variations in the corresponding NTCP for the endpoints under consideration. The commissioning of the new available version of our TPS (Raystation v.10B), which introduces GPU-based Monte Carlo dose engine for optimization and dose calculations of PT plans, will allow the re-optimization of IMPT plans using a robust approach on multiple scenarios of uncertainties and possibly confirm our statement.

For proton plans, larger uncertainties in RBE modeling are expected at the distal end of the Bragg Peak [25]. OAR with serial architecture in the H&N district, e.g., optical pathways, brainstem, and cord, might be affected by potential higher toxicities when located distally to the target. While tumor is generally located away from the optical pathways in the cranio-caudal direction, beam geometry was defined so that brainstem and cord are intersected in the transversal plane only by the lateral penumbra for the two contralateral beams, thus mitigating the possible impact on biological dose.

Another limitation of our study is related to the NTCP model selection. The majority of the NTCP models used lacks internal and/or external validation with an acceptable level of evidence [27]. Some models were validated for different patient cohorts and treatment techniques, thus introducing uncertainties in the estimated toxicity rates. However, we expect that the application of alternative models might lead to different absolute NTCP values while preserving the general trend in the relative difference between proton ad photon plans. Of the seven NTCP models selected for the MBS methodology, the ones having the most substantial impact on our results (xerostomia and brain necrosis) were validated for PT. The photon-derived model for acute mucositis was proved to be valid for proton therapy patients [49], while the one predicting trismus was already used in comparative studies between proton and photon plans in HNC patients [50].

Finally, future toxicity evaluations focused on NPC patients should also consider the risk of relevant, although rare, late side effects such as hypoglossal nerve palsy, and ischemic stroke related to carotid artery stenosis [5].The use of additional NTCP models relative to the same endpoint, as suggested by Brodin et al. [51], together with estimations of uncertainties in target contouring, treatment planning and delivery of PT and uncertainties in RBE modeling may increase the robustness of our conclusions.

## 5. Conclusions

The use of NTCP models for predicting toxicity risk may become a gold standard in the near future for evaluating the best treatment option in RT. We implemented an integrated strategy with two working pipelines to support clinical NPC patients allocation between proton and photon radiotherapy. The introduction of a new synthetic index (i.e., CTS) for describing the overall expected reduction in OARs toxicities with PT was supported by the application of the well-established MBS methodology on the same patient cohort. IMPT plans provided a substantial reduction in dose-volume evaluators and NTCP values in almost half of the examined population, reflecting an expected major benefit in patient QOL. Further clinical validation of NTCP-based predictions is mandatory to confirm the robustness of model-based approach in clinical practice.

## Figures and Tables

**Table 1:** Details of the NTCP models set, MBS threshold values and CTS weights. For the 7 models selected for MBS analysis, corresponding threshold values. CTS weights were defined following a clinical rationale. In particular, the degree of impact on the patient QOL for a specific toxicity translate to a higher relative weight. Weight values sum up to unity. Dysphagia, xerostomia, and trismus yielded the higher relative weight values. NTCP models referring to similar endpoints had a small relative value (0.005).

NTCP Model			MBS	MBS Thresholds		CTS Weights $w_{i}$
Author	Organ	Endpoint (Time post-RT)		Young	Standard	
Niyazi (2020) [30]	Brain	Necrosis > grade II (2 years)	yes	5%	10%	0.1
Kong (2016) [34]	Temporal Lobe	Temporal Lobe Infarction (>3 months)				0.1
Palorini (2019) [35]	Optic Pathways	Radiation Induced Ocular Toxicity (RION) (>3 months)	yes	3%	5%	0.07
Palorini (2019) [35]	Optic Pathways	Grade IV Visual Acuity Loss (>3 months)	yes	3%	5%	0.07
Rancati (2009) [36]	Glottic	Edema grade II (15 months)				0.005
Eisbruch (2011) [37]	Glottic	Aspiration (12 months)				0.005
Bhide (2012) [38]	Oral Cavity	Mucositis (8 weeks)	yes	10%	10%	0.05
Eisbruch (2011) [37]	Superior PCM	Aspiration (12 months)				0.005
Loizeau (2021) [31]	Superior PCM	Grade II-IV dysphagia (6 months)	yes	3%	5%	0.2
Christianen (2012) [39]	Superior PCM	Problems swallowing solids (6 months)				0.005
Christianen (2012) [39]	Superior PCM	Problems swallowing liquids (6 months)				0.005
Loizeau (2021) [31]	Parotid	Moderate to severe xerostomia (6 months)	yes	15%	15%	0.2
Roesink (2001) [40]	Parotid	Flow ratio < 25% (1 year)				0.005
Lee (2015) [41]	Cochlea	Tinnitus (2 years)				0.01
Lindblom (2014) [33]	TMJ	Trismus (>3 months)		7.5%	10%	0.15
Vogelius (2011) [42]	Thyroid	Hypothyroidism (2 years)				0.02

**Table 2:** Clinical and treatment–related characteristics of the 50 patients included in the study.

		Total	Percentage (%)
All patients		50	100
Histology	Keratinizing squamous cell carcinoma (WHO type 1)	3	6
	Undifferentiated (WHO type 2)	47	94
Stage T	1	20	40.0
	2	7	14.0
	3	15	30.0
	4	8	16.0
Stage N	0	8	16.0
	1	14	28.0
	2	15	30.0
	3	13	26.0
Stage	I	2	4.0
	II	7	14.0
	III	19	38.0
	IVA	10	20.0
	IVB	12	24.0
Treatment	RT alone	4	8.0
	RT-CHT	27	54.0
	iCHT + RT-CHT	19	38.0
Target Volume (cc) [mean ± standard deviation]	PTV HD	239.5 ± 111.2	
	PTV ID	447.1 ± 200.5	
	PTV LD	612.2 ± 129.9	

**Table 3:** The percentage of patients eligible for PT with MBS strategy with the standard and the “young” threshold values. In addition, passing rates applying the single or the composite threshold and for each NTCP model were reported. Values were shown relative to each subgroup of the cohort stratification.

Classification	All Patients		Tumor Staging		Nodal Involvement		
Subgroups	Adult	Young	T1T2	T3T4	N0	N1	N2N3
Number of patients	*n* = 50		*n* = 27	*n* = 23	*n* = 8	*n* = 14	*n* = 28
PT eligibility							
Standard	40.0%		25.9%	54.2%	12.5%	42.9%	46.4%
Young	84.0%		79.9%	89.1%	61.3%	84.0%	90.7%
Passing rates							
for threshold:							
Single	36.0%	84.0%	22.2%	50.0%	12.5%	42.9%	39.3%
Composite	20.0%	18.0%	11.1%	29.2%	12.5%	21.4%	21.4%
for each model:							
Brain Necrosis > G2 (2 years)	8.0%	46.0%	3.7%	12.5%	0.0%	14.3%	7.1%
RION/G4 Visual Acuity Loss (>3 months)	0.0%	0.0%	0.0%	0.0%	0.0%	0.0%	0.0%
Mucositis (8 weeks)	6.0%	6.0%	0.0%	12.5%	12.5%	7.1%	3.6%
Dysphagia (6 months)	2.0%	12.0%	0.0%	4.2%	12.5%	0.0%	0.0%
Xerostomia (1 year)	24.0%	66.0%	18.5%	29.2%	0.0%	14.3%	35.7%
Trismus (>3 months)	6.0%	6.0%	7.4%	4.2%	0.0%	7.1%	7.1%

## Data Availability

The data presented in this study are available on request from the corresponding author.

## Supplementary Materials

The following supporting information can be downloaded at: https://www.mdpi.com/article/10.3390/cancers14051109/s1, Table S1: Dose volume points for IMPT and VMAT plans and relative difference; Table S2: Homogeneity and conformity index for IMPT and VMAT plans and relative difference; Table S3: Dose-volume points for a list of significant OAR for NPC patients. The dose-constraint used for plan optimization (internal protocol) is reported below each OAR. Table S4: Calculated CTS values for the different groups. Figure S1: NTCP values for the 16 models for the full patient cohort (a); patients eligible for PT (b); patients not eligible for PT (c).
