# Supplementary material for: Proton Radiation Therapy for Nasopharyngeal Cancer Patients: Dosimetric and NTCP Evaluation Supporting Clinical Decision
Source: Cancers (Basel). 2022 Feb 22;14(5):1109. doi: 10.3390/cancers14051109 (PMC8909055; doi:10.3390/cancers14051109)
Supplement: Supplementary file 1 [file cancers-14-01109-s001.zip › cancers-1570979-supplementary.pdf]

Supplementary Materials

Table S1: Dose volume points for IMPT and VMAT plans and relative difference.

	Protons [Gy(RBE)]			Protons [Gy(RBE)]			Δ p-X [Gy(RBE)]	Δ p-X [%]
	**PTV_{HD}**							
**$D_{99\%}$**	67,4	±	1,2	67,8	±	13,1	-0,4	-0,6
**$D_{98\%}$**	68,1	±	0,5	68,2	±	0,9	-0,1	-0,1
**$D_{95\%}$**	68,9	±	0,3	68,7	±	0,5	0,2	0,3
**$D_{50\%}$**	70,5	±	0,6	70,4	±	0,3	0,1	0,1
**$D_{2\%}$**	71,9	±	0,7	72,5	±	0,5	-0,6	-0,8
**$D_{1\%}$**	72,2	±	0,7	72,7	±	0,5	-0,6	-0,8
**D_{mean}**	70,4	±	0,5	70,4	±	0,3	0,0	0,0
**D_{min}**	55,8	±	7,8	57,7	±	11,1	-1,9	-3,3
**D_{max}**	74,6	±	1,4	74,7	±	1,2	-0,1	-0,1
	**$PTV_{ID\_1,8Gy(RBE)}$**							
**$D_{99\%}$**	58,3	±	1,7	59,1	±	2,6	-0,9	-1,5
**$D_{98\%}$**	58,9	±	1,7	59,5	±	2,6	-0,6	-1,0
**$D_{95\%}$**	59,8	±	1,8	60,1	±	2,6	-0,4	-0,6
**$D_{50\%}$**	69,1	±	2,7	69,0	±	2,6	0,0	0,0
**$D_{2\%}$**	71,8	±	0,8	72,2	±	0,4	-0,4	-0,6
**$D_{1\%}$**	72,0	±	0,8	72,5	±	0,5	-0,5	-0,7
**D_{mean}**	67,4	±	1,8	67,7	±	1,8	-0,3	-0,5

	Protons [Gy(RBE)]			Protons [Gy(RBE)]			Δ p-X [Gy(RBE)]	Δ p-X [%]
	$PTV_{LD\_1,8-2,1Gy(RBE)}$							
$D_{99\%}$	57,2	±	2,4	58,6	±	2,6	-1,4	-2,4
$D_{98\%}$	57,9	±	2,1	58,9	±	2,5	-1,0	-1,7
$D_{95\%}$	58,9	±	1,7	59,4	±	2,4	-0,6	-0,9
$D_{50\%}$	62,8	±	2,3	63,6	±	3,1	-0,8	-1,2
$D_{2\%}$	70,0	±	1,6	70,8	±	1,9	-0,8	-1,2
$D_{1\%}$	70,5	±	1,4	71,3	±	1,7	-0,8	-1,1
D_{mean}	63,4	±	1,9	64,2	±	2,2	-0,8	-1,3
D_{min}	43,8	±	11,2	39,3	±	18,7	4,5	11,4
D_{max}	74,2	±	1,6	74,4	±	1,8	-0,3	-0,3
	$PTV_{LD\_1,7-2,1Gy(RBE)}$							
$D_{99\%}$	52,8	±	1,0	52,9	±	5,7	-0,1	-0,3
$D_{98\%}$	53,4	±	0,9	53,8	±	3,8	-0,4	-0,8
$D_{95\%}$	54,2	±	0,9	55,3	±	0,3	-1,1	-2,0
$D_{50\%}$	56,7	±	1,1	57,4	±	0,8	-0,7	-1,3
$D_{2\%}$	68,6	±	3,5	68,9	±	3,7	-0,3	-0,4
$D_{1\%}$	69,5	±	2,8	69,7	±	3,1	-0,3	-0,4
D_{mean}	58,5	±	1,9	59,3	±	1,7	-0,7	-1,2

**D_{min}**	43,7	±	12,4	39,6	±	19,3	4,0	10,2		D_{min}	35,9	±	6,1	33,6	±	14,0	2,4	7,0
**D_{max}**	74,9	±	1,6	74,9	±	1,3	0,1	0,1		D_{max}	74,5	±	2,3	73,8	±	1,3	0,7	1,0
**$PTV_{LD\_1,7Gy(RBE)}$**										$PTV_{LD\_1,7-1,8Gy(RBE)}$								
**$D_{99\%}$**	54,4	±	1,2	54,8	±	2,7	-0,3	-0,6		$D_{99\%}$	53,1	±	1,2	54,1	±	3,2	-1,0	-1,8
**$D_{98\%}$**	55,4	±	1,2	55,6	±	1,0	-0,2	-0,4		$D_{98\%}$	54,4	±	0,7	54,9	±	2,2	-0,4	-0,8
**$D_{95\%}$**	56,2	±	1,3	56,2	±	1,1	0,0	0,0		$D_{95\%}$	55,9	±	0,4	55,7	±	1,1	0,2	0,4
**$D_{50\%}$**	63,9	±	4,8	64,8	±	4,8	-1,0	-1,5		$D_{50\%}$	58,3	±	1,2	58,5	±	2,0	-0,1	-0,2
**$D_{2\%}$**	71,6	±	0,8	72,0	±	0,4	-0,4	-0,5		$D_{2\%}$	66,3	±	1,6	69,0	±	1,4	-2,7	-3,9
**$D_{1\%}$**	71,9	±	0,8	72,4	±	0,4	-0,5	-0,7		$D_{1\%}$	67,5	±	1,4	69,8	±	1,2	-2,3	-3,3
**D_{mean}**	63,9	±	2,4	64,4	±	2,2	-0,5	-0,7		D_{mean}	58,9	±	1,2	59,6	±	1,7	-0,8	-1,3
**D_{min}**	34,5	±	8,0	30,4	±	17,0	4,1	13,4		D_{min}	34,5	±	8,4	27,1	±	18,5	7,4	27,4
**D_{max}**	75,1	±	2,1	74,8	±	1,3	0,3	0,4		D_{max}	73,5	±	2,4	74,4	±	1,8	-0,8	-1,1

Table S2: Homogeneity and conformity index for IMPT and VMAT plans and relative difference.

	**Protons**			**Photons**			**p-X**	
**PTV_{HD}**	Mean			Mean			% Δ	*p-value*
**HI**	0,05	±	0,01	0,06	±	0,01	-12%	0,1277
**CI**	1,30	±	0,10	1,46	±	0,16	-11%	0
**$PTV_{ID\_1,8Gy(RBE)}$**								
**HI**	0,19	±	0,03	0,18	±	0,04	2%	0,2838
**CI**	1,86	±	0,47	1,97	±	0,62	-6%	0,0063
**$PTV_{LD\_1,7Gy(RBE)}$**								
**HI**	0,26	±	0,03	0,26	±	0,03	0%	0,9029
**CI**	1,45	±	0,09	1,66	±	0,35	-13%	0
**$PTV_{LD\_1,8-2,1Gy(RBE)}$**								
**HI**	0,19	±	0,03	0,19	±	0,04	2%	0,3261
**CI**	4,90	±	2,00	5,21	±	2,29	-6%	0,0044
**$PTV_{LD\_1,7-1,8Gy(RBE)}$**								
**HI**	0,27	±	0,07	0,26	±	0,10	2%	0,6071
**CI**	2,74	±	0,88	3,35	±	1,53	-20%	0,001
**$PTV_{LD\_1,7-1,8Gy(RBE)}$**								
**HI**	0,20	±	0,03	0,24	±	0,05	-18%	0,0439
**CI**	4,21	±	1,98	4,69	±	2,32	-11%	0,0001

Table S3: Dose-volume points for a list of significant OAR for NPC patients. The dose-constraint used for plan optimization (internal protocol) is reported below each OAR.

			p [GyE]		X [GyE]		Δ(p-X)	
			Median	IR	Median	IR	[GyE]	[%]
**Temp.Lobe R**		D99	0,1	0,0	2,1	1,2	-2,0	-97,1
**D01< 65 Gy**		D50	0,5	1,1	9,3	6,5	-8,8	-95,0
		D02	53,1	12,7	50,2	13,5	3,0	5,9
		D01	59,0	9,8	57,1	13,1	2,0	3,5
		DoseMax	70,6	4,7	69,9	5,4	0,7	1,0
		DoseAverage	7,4	5,1	13,0	6,5	-5,6	-43,1
		DoseMin	0,1	0,0	1,7	1,1	-1,7	-97,1
**Temp.Lobe L**		D99	0,1	0,1	2,1	1,1	-2,0	-96,6
**D01< 65 Gy**		D50	0,6	1,6	8,1	7,1	-7,4	-92,1
		D02	51,9	23,7	52,6	22,0	-0,7	-1,4
		D01	56,1	18,9	58,6	18,6	-2,5	-4,2
		DoseMax	70,8	6,3	70,6	7,0	0,2	0,2
		DoseAverage	7,5	7,2	12,3	7,5	-4,9	-39,5
		DoseMin	0,1	0,1	1,8	0,9	-1,8	-96,4
**Brain**		D99	0,0	0,1	0,5	0,2	-0,5	-97,8
**D01< 65 Gy**		D50	0,1	0,0	2,3	1,3	-2,1	-94,7
		D02	26,7	23,3	40,3	12,3	-13,6	-33,8
		D01	38,9	24,9	45,9	13,7	-7,1	-15,4
		DoseMax	72,6	2,8	72,6	2,3	0,0	0,0
		DoseAverage	1,5	1,5	7,5	3,5	-6,0	-80,0
		DoseMin	0,0	0,0	0,4	0,1	-0,4	-97,4
**Brainstem**		D99	0,2	0,1	7,3	7,6	-7,1	-97,3
**D01< 54 Gy**		D50	6,0	5,6	30,2	11,0	-24,2	-80,2
		D02	45,6	15,4	48,9	5,7	-3,3	-6,8
		D01	49,0	13,9	50,2	5,3	-1,2	-2,4
		DoseMax	60,4	12,6	55,7	6,2	4,8	8,6
		DoseAverage	11,4	7,2	28,4	9,2	-16,9	-59,7
		DoseMin	0,1	0,1	6,9	6,1	-6,8	-98,2
**Spinal Cord**		D99	0,0	0,0	2,1	9,3	-2,1	-99,5
**D01< 45 Gy**		D50	0,2	0,4	30,0	3,9	-29,8	-99,3
		D02	11,4	10,9	35,8	3,6	-24,4	-68,1
		D01	14,0	13,7	36,1	3,9	-22,1	-61,2
		DoseMax	25,9	18,3	37,8	4,8	-11,9	-31,4
		DoseAverage	1,8	1,4	25,7	4,7	-23,9	-93,1
		DoseMin	0,0	0,0	2,0	8,3	-2,0	-99,5

**Opt.Nerve L**	D99	0,9	1,0	4,8	3,0	-3,8	-80,9
**D01< 55 Gy**	D50	6,6	9,8	8,9	9,0	-2,3	-26,1
	D02	20,3	21,9	19,8	24,7	0,5	2,6
	D01	21,0	22,2	20,5	24,4	0,6	2,7
	DoseMax	23,8	23,3	21,1	23,9	2,7	12,9
	DoseAverage	7,4	10,1	10,1	11,4	-2,8	-27,4
	DoseMin	0,8	0,8	4,5	2,9	-3,8	-83,4
**Opt.Nerve R**	D99	0,9	0,8	4,6	2,9	-3,7	-80,6
**D01< 55 Gy**	D50	6,2	5,9	9,0	6,8	-2,8	-31,3
	D02	18,8	20,0	19,0	20,4	-0,2	-1,2
	D01	19,8	21,2	19,5	21,1	0,3	1,4
	DoseMax	21,5	23,4	21,0	22,4	0,5	2,2
	DoseAverage	7,2	7,2	10,2	6,9	-3,1	-29,8
	DoseMin	0,8	0,7	4,5	2,8	-3,8	-83,4
**Optic Chiasm**	D99	1,2	2,9	5,3	3,3	-4,1	-77,4
**D01< 55 Gy**	D50	4,7	10,5	7,2	9,8	-2,5	-35,1
	D02	11,3	23,1	10,4	29,6	0,9	8,2
	D01	11,7	24,7	10,6	29,9	1,1	10,2
	DoseMax	14,4	31,5	11,0	29,8	3,4	30,8
	DoseAverage	5,3	10,6	7,6	13,1	-2,3	-30,3
	DoseMin	1,1	2,8	5,2	3,2	-4,2	-79,6
**Supraglottic Larynx**	D99	17,4	19,3	31,6	10,6	-14,3	-45,2
**$D_{average}$ < 55 Gy**	D50	41,5	12,2	41,8	8,2	-0,4	-0,9
	D02	59,2	7,9	59,9	7,7	-0,7	-1,2
	D01	60,8	8,9	62,1	8,0	-1,3	-2,1
	DoseMax	67,3	10,9	67,1	8,8	0,2	0,3
	DoseAverage	40,2	9,1	43,4	8,7	-3,2	-7,3
	DoseMin	13,0	19,2	30,5	10,6	-17,5	-57,4
**Glottic Larynx**	D99	10,9	11,4	28,9	8,1	-18,1	-62,5
**$D_{average}$ < 45 Gy**	D50	20,8	14,1	35,2	7,1	-14,4	-40,8
	D02	40,4	13,9	46,3	9,4	-6,0	-12,9
	D01	43,3	13,5	47,2	9,6	-3,9	-8,2
	DoseMax	50,8	12,9	52,0	9,9	-1,2	-2,3
	DoseAverage	21,9	13,0	35,9	8,1	-13,9	-38,8
	DoseMin	9,7	11,7	28,2	8,6	-18,5	-65,7
**Oral Cavity**	D99	1,5	1,9	20,7	5,7	-19,2	-92,9
**$D_{average}$ < 40 Gy**	D50	38,2	10,1	34,8	6,2	3,4	9,6
	D02	69,8	3,7	69,7	2,8	0,1	0,1
	D01	70,4	2,0	70,6	2,1	-0,2	-0,3
	DoseMax	72,0	1,2	72,3	0,9	-0,3	-0,4

		DoseAverage	36,5	6,8	39,3	4,9	-2,8	-7,1
		DoseMin	0,6	0,6	19,3	5,9	-18,7	-96,7
**Sup PCM**		D99	57,7	10,1	57,6	9,6	0,1	0,2
**$D_{average}$ < 50 Gy**		D50	69,6	6,1	69,4	2,9	0,2	0,3
		D02	71,4	1,2	71,8	1,0	-0,4	-0,6
		D01	71,6	1,3	71,9	1,0	-0,3	-0,5
		DoseMax	72,3	1,4	72,5	1,1	-0,2	-0,3
		DoseAverage	67,8	5,3	67,8	4,8	0,0	0,0
		DoseMin	54,7	10,1	54,6	10,3	0,2	0,3
**Mid PCM**		D99	33,3	18,6	48,8	8,6	-15,6	-31,9
**$D_{average}$ < 50 Gy**		D50	57,7	9,9	59,6	11,8	-1,9	-3,3
		D02	70,3	7,6	71,1	5,7	-0,8	-1,1
		D01	70,9	5,6	71,3	3,2	-0,5	-0,7
		DoseMax	71,9	2,4	72,3	2,2	-0,4	-0,6
		DoseAverage	56,1	9,8	60,4	8,7	-4,3	-7,1
		DoseMin	29,3	18,8	47,1	8,3	-17,8	-37,8
**Inf PCM**		D99	4,8	11,0	41,6	7,5	-36,8	-88,5
**$D_{average}$ < 50 Gy**		D50	35,5	17,4	48,1	7,2	-12,6	-26,2
		D02	57,0	8,4	59,0	9,2	-2,1	-3,5
		D01	58,3	9,7	60,1	11,2	-1,9	-3,1
		DoseMax	62,9	11,1	65,2	12,0	-2,3	-3,6
		DoseAverage	33,1	14,3	49,2	7,0	-16,1	-32,7
		DoseMin	3,1	8,1	40,6	7,1	-37,5	-92,3
**Esophagus**		D99	0,5	1,9	29,8	18,1	-29,3	-98,2
**$D_{average}$ < 35 Gy**		D50	15,7	18,0	40,2	10,5	-24,6	-61,0
**V_{45Gy} <= 33%**		D02	46,0	20,0	50,2	9,1	-4,2	-8,4
**V_{54Gy} <= 15%**		D01	48,8	19,7	51,1	9,0	-2,4	-4,6
		DoseMax	53,4	18,9	53,9	7,3	-0,5	-1,0
		DoseAverage	19,3	12,5	39,3	10,5	-20,0	-50,9
		DoseMin	0,3	1,1	29,1	19,3	-28,9	-99,1
**Parotid R**		D99	7,7	3,4	20,9	7,7	-13,2	-63,2
**$D_{average}$ < 26 Gy**		D50	24,8	5,0	37,8	11,3	-13,0	-34,3
**V_{30Gy} <= 50%**		D02	65,9	13,8	68,4	12,0	-2,5	-3,6
		D01	68,1	13,1	69,5	11,3	-1,4	-2,0
		DoseMax	71,3	8,8	71,7	6,1	-0,4	-0,6
		DoseAverage	29,6	5,7	41,3	9,6	-11,7	-28,4
		DoseMin	6,3	3,1	17,2	7,2	-10,9	-63,6
**Parotid L**		D99	7,6	3,6	24,3	9,8	-16,7	-68,7
**$D_{average}$ < 26 Gy**		D50	25,5	8,6	41,3	21,7	-15,8	-38,2
**V_{30Gy} <= 50%**		D02	69,5	11,5	70,1	10,0	-0,6	-0,8

		D01	70,2	11,1	70,6	8,0	-0,4	-0,5
		DoseMax	71,7	3,3	72,0	1,9	-0,3	-0,5
		DoseAverage	31,3	8,3	43,8	14,0	-12,5	-28,5
		DoseMin	6,1	3,8	20,4	8,9	-14,3	-70,2
**Cochlea R**		D99	17,9	16,1	23,8	14,2	-5,9	-24,9
**$D_{average}$ < 35-45 Gy**		D50	30,1	15,8	31,2	12,8	-1,1	-3,4
		D02	46,8	17,4	45,5	19,7	1,4	3,0
		D01	48,1	17,9	46,7	19,5	1,4	3,0
		DoseMax	51,7	19,6	47,9	21,3	3,8	7,9
		DoseAverage	30,7	15,7	32,4	12,8	-1,8	-5,5
		DoseMin	15,3	15,1	23,8	14,2	-8,5	-35,7
**Cochlea L**		D99	14,0	19,1	24,2	17,6	-10,2	-42,1
**$D_{average}$ < 35-45 Gy**		D50	26,1	26,9	32,6	20,0	-6,5	-19,9
		D02	45,3	27,7	49,0	28,6	-3,7	-7,6
		D01	47,0	27,5	50,6	29,3	-3,6	-7,2
		DoseMax	51,0	26,4	52,5	30,7	-1,5	-2,8
		DoseAverage	26,8	26,2	33,0	19,7	-6,2	-18,9
		DoseMin	12,7	19,4	24,4	17,0	-11,8	-48,1
**Thyroid**		D99	21,9	14,7	36,9	12,8	-15,0	-40,6
**$D_{average}$ < 45 Gy**		D50	48,8	3,3	54,3	3,1	-5,5	-10,1
		D02	60,0	8,2	60,8	10,1	-0,8	-1,3
		D01	60,7	10,4	61,4	11,1	-0,8	-1,2
		DoseMax	64,0	11,1	62,9	11,9	1,1	1,7
		DoseAverage	46,8	3,6	52,5	5,1	-5,8	-11,0
		DoseMin	11,9	16,9	33,9	14,1	-22,0	-64,8
**Mandible**		D99	1,0	2,5	21,8	7,7	-20,8	-95,6
**D01 < 70 Gy**		D50	27,1	9,4	39,4	7,2	-12,3	-31,3
		D02	60,6	12,7	64,8	9,6	-4,3	-6,6
		D01	65,1	12,4	66,7	8,8	-1,6	-2,4
		DoseMax	70,9	5,2	71,4	2,4	-0,5	-0,7
		DoseAverage	28,7	8,4	40,4	5,6	-11,8	-29,1
		DoseMin	0,7	0,5	18,8	6,6	-18,2	-96,5

Table S4: Calculated CTS values for the different groups.

	ALL (50 pts)	***N0 (8 pts)***	***N1 (14 pts)***	***N2N3 (28 pts)***	***T1T2 (27 pts)***	***T3T4 (23 pts)***
**Eligible**	6.3 ± 1.4	6.8 ± 0	6.4 ± 0.5	6.3 ± 1.8	6.8 ± 1.1	6.1 ± 1.6
**Not Eligible**	3.5 ± 1.4	3.3 ± 1.7	3.3 ± 0.9	3.6 ±1.6	3.2 ±1.3	4.2 ± 1.6

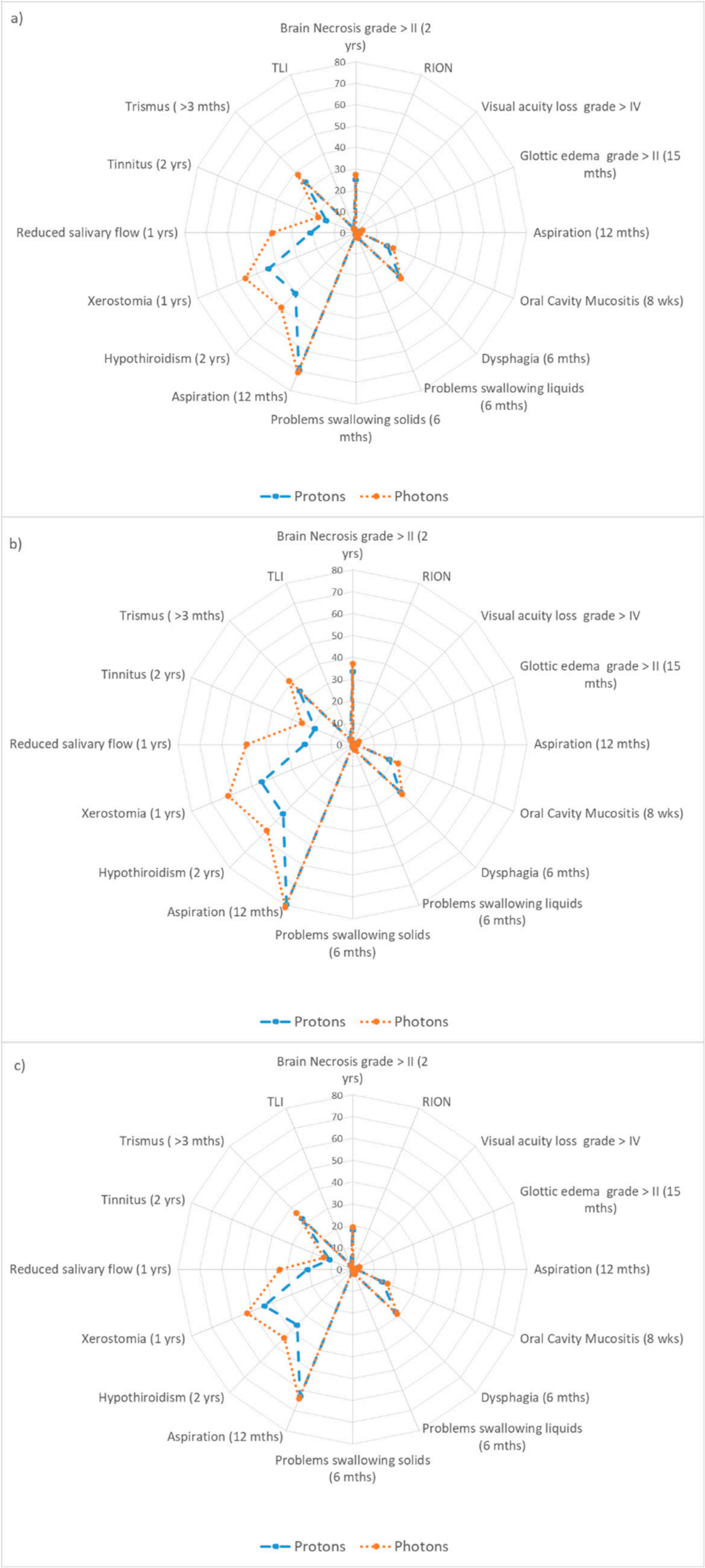

Figure S1: NTCP values for the 16 models for the full patient cohort (a); patients eligible for PT (b); patients not eligible for PT (c).
